# Therapeutic effects of non-invasive, individualized, transcranial neuromodulation treatment for voiding dysfunction in multiple sclerosis patients: study protocol for a pilot clinical trial

**DOI:** 10.1186/s40814-021-00825-z

**Published:** 2021-03-24

**Authors:** Khue Tran, Zhaoyue Shi, Christof Karmonik, Blessy John, Hamida Rajab, Santosh A. Helekar, Timothy Boone, Rose Khavari

**Affiliations:** 1 Department of Urology, Houston Methodist Hospital, 6560 Fannin St. Suite 2100, Houston, TX 77030 USA; 2 Translational Imaging Center, Houston Methodist Research Institute, Houston, TX USA; 3 Department of Neurology, Houston Methodist Hospital, Houston, TX USA; 4 Department of Neurosurgery, Houston Methodist Hospital, Houston, TX USA

**Keywords:** Multiple sclerosis, Voiding dysfunction, fMRI, Neuromodulation, Urodynamics, Urinary retention

## Abstract

**Background:**

Voiding dysfunction (VD) is a common neurogenic lower urinary tract dysfunction (NLUTD) in multiple sclerosis (MS) patients. Currently, the only effective management for VD and urinary retention in MS patients is catheterization, prompting us to look for novel therapeutic options beyond the bladder, such as the brain. Transcranial rotating permanent magnet stimulator (TRPMS) is a non-invasive, portable, multifocal neuromodulator that simultaneously modulates multiple cortical regions, enhancing or attenuating strengths of functional connections between these regions. The objective of this pilot clinical trial is to evaluate the feasibility of a TRPMS trial to address lower urinary tract symptoms in MS patients, through investigating the therapeutic effects of TRPMS in modulating brain regions during voiding initiation and mitigating VD in female MS individuals.

**Methods:**

Ten adult female MS patients with VD (defined as having %post-void residual/bladder capacity (%PVR/BC) ≥ 40% or Liverpool nomogram percentile < 10%) will be recruited for this study. Concurrent urodynamic and functional MRI evaluation with a bladder filling/emptying task repeated three to four times will be performed at baseline and post-treatment. Predetermined regions of interest and their blood-oxygen-level-dependent (BOLD) activation at voiding initiation will be identified on each patient’s baseline anatomical and functional MRI scan, corresponding to the microstimulators placement on their individualized TRPMS treatment cap to either stimulate or inhibit these regions. Patients will receive 10 40-min treatment sessions. Non-instrumented uroflow and validated questionnaires will also be collected at baseline and post-treatment to evaluate clinical improvement.

**Discussion:**

Despite the crucial role of the central nervous system in urinary control and its sensitivity to MS, there has been no treatment for urinary dysfunction targeting the brain centers that are involved in proper bladder function. This trial, to our knowledge, will be the first of its kind in humans to consider non-invasive and individualized cortical modulation for treating VD in MS patients. Results from this study will provide a better understanding of the brain control of neurogenic bladders and lay the foundation for a potential alternative therapy for VD in MS patients and other NLUTD in a larger neurogenic population in the future.

**Trial registration:**

This trial is registered at ClinicalTrials.Gov (NCT03574610, 2 July 2018.) and Houston Methodist Research Institute IRB (PRO00019329)

## Background

Multiple sclerosis (MS) is a chronic multifocal demyelinating disease that can affect any part of the central nervous system. Due to the heterogeneity of MS symptoms and severity, the impact on quality of life can vary for each patient. However, urinary symptoms are one of the most common manifestations of MS, with up to 90% of MS patients experiencing some degree of voiding dysfunction (VD) and/or incontinence in their life [1]. VD is a morbid and costly urological condition characterized by intermittent, fluctuating, or absent flow of urine [2]. VD may lead to incomplete bladder emptying or urinary retention, urinary tract infections, sepsis, bladder or kidney stones, and permanent renal failure [3]. Currently, the only available therapies for VD include indwelling bladder catheters or intermittent self-catheterizations. Catheterization is a burden, in general, and even more so in neuropathic patients, in whom lower extremity spasms, compromised hand dexterity, or visual disturbances, may be present. The cost and morbid side effects (hematuria, pain, trauma, strictures, and infections) associated with catheterizations prompts us to look into alternate therapeutic targets for VD, beyond the bladder, such as the brain where bladder control centers are located.

Transcranial magnetic stimulation (TMS) is a non-invasive centrally acting electromagnetic neuromodulation in which an electromagnetic coil is held over the scalp to deliver a rapidly pulsed magnetic field to the cortex to activate (modulate) neurons, within a limited area, without requiring anesthesia or causing any significant side effects. Two studies have demonstrated the feasibility and efficacy of repetitive TMS in patients with MS (*n* = 10) and Parkinson’s disease (*n* = 8) with bladder symptoms. Their findings suggest that TMS improves storage phase symptoms in Parkinson’s disease and voiding phase symptoms in MS, reduces urinary post-void residual, while appropriately increasing the detrusor pressure at the time of voiding [4, 5]. Despite its potential, limitations still exist with TMS. Commercialized TMS devices use large coils that restrict their portability and can only be applied to one location of the cortex. However, brain control during the storage phase is dependent on multiple regions and their connectivity, requiring a transcranial modulator that could safely stimulate/inhibit multiple cortical areas at the same time.

The Neurophysiology and Neuromodulation Laboratory at Houston Methodist Hospital has developed a non-invasive, portable, and multifocal brain magnetic modulator, called Transcranial Rotating Permanent Magnet Stimulator (TRPMS) [6]. TRPMS is a wearable and portable cortical neuromodulation device with multifocal stimulation, using small, high-field strength permanent magnets rapidly spun by miniature electric motors [7]. Compared to conventional TMS, TRPMS might enable greater uniformity, consistency, and focality in the stimulation of targeted cortical areas subject to significant anatomical variability. For this pilot clinical trial, we propose to explore the therapeutic effects of TRPMS in mitigating VD symptoms in MS patients.

In a small group of women with urinary retention, due to urethral sphincter dysfunction, peripheral nerve neuromodulation modified the activity of the right post-central gyrus, precentral/temporal, and inferior temporal regions [8, 9]. Functional magnetic resonance imaging (fMRI) studies report that medial prefrontal cortex and supplementary motor areas are the main cortical regions involved in pelvic floor contraction [10–12], and could be used to phenotype responders versus non-responders to biofeedback-assisted pelvic floor physical therapy in overactive bladder patients [13]. Earlier neuroimaging studies have also identified brain regions directly involved in initiating or continuing voiding in healthy individuals. These regions include precentral gyrus, supplementary motor area, dorsolateral prefrontal lobe, inferior frontal gyrus (IFG), cingulate gyrus, insula, hypothalamus, periaqueductal grey (PAG), and pons (pontine micturition center (PMC)) [14–17]. Although PAG and PMC are the more apparent regions involved in initiation of voiding and could serve as potential targets for intervention, they are deep and inaccessible with our current technological modalities. Besides, these regions are responsible for other core vital functions such as regulating circulation and breathing wherein modulation would not be safe. On the other hand, we propose that if we shift our focus to modulate areas of the brain that are more cortically accessible and amendable to modulation, such as areas responsible for urethral sphincter relaxation, we may indirectly improve voiding.

Therefore, for this pilot study, we hypothesize that individualized, non-invasive, multifocal, targeted cortical modulation in MS patients using TRPMS can produce changes in brain activation at voiding initiation that better recapitulate the activation patterns in healthy individuals, leading to clinical improvement in VD symptoms in this patient population. Additionally, we propose to include the modulation of regions involved in depression, to potentially mitigate the psychological consequences of depression seen in many MS patients [18] in addition to regions involved in anxiety, both of which could affect patients’ ability to initiate voiding [19, 20]. The protocol and results from this pilot study will determine the feasibility and provide the foundation for future TRPMS trials addressing storage symptoms in addition to VD in MS patients.

## Methods and design

### Study design

This is a hypothesis driven, prospective, single-center, open-label pilot clinical trial evaluating the effectiveness of individualized, non-invasive TRPMS treatment to modulate (stimulate/inhibit) specific cortical regions in MS patients with VD. All participants will receive 10 TRPMS treatments session. Their neuroimaging and clinical data will be assessed and compared at baseline and post-treatment to assess the therapeutic effects of individualized, non-invasive, cortical modulation using TRPMS in improving VD symptoms.

### Study objectives

The primary objective of this study is to determine the feasibility of TRPMS trials for improving lower urinary tract symptoms in female MS patients, through the following outcomes: recruitment rate, retention rate, and expected effect size.

The secondary objective is to assess the therapeutic effects of TRPMS through three study outcomes: (1) brain activation and connectivity via the blood-oxygen-level-dependent (BOLD) signals and functional connectivity (FC) in modulated regions and regions of interest (ROI) following TRPMS treatment; (2) voiding improvement via urodynamic (UDS) parameters and validated questionnaires regarding to participants’ bladder symptoms and anxiety/depression following TRPMS treatment; and (3) baseline clinical, UDS, and/or neuroimaging factors that could predict response to TRPMS treatment.

### Study population and recruitment

Female MS patients will be recruited from the Houston Methodist Neurourology clinic. Additionally, recruitment flyers will be posted in our Urology and Neurourology for patients who are interested. Adult patients ≥ 18 years of age with clinically stable MS (Expanded Disability Status Score (EDSS) ≤ 6.5) and symptomatic NLUTD will be screened. Patients with VD (defined as having %PVR/bladder capacity (BC) ≥ 40%; or having Liverpool Nomogram percentile < 10%; or performing self-catheterization) will be invited to participate.

Men will be excluded from this current study to avoid confounding by prostatic pathology. Other exclusion criteria include severe debilitating disease, pregnancy or planning to become pregnant, nursing, contraindications to MRI, history of interstitial cystitis, augmentation cystoplasty, presence of other neurological diseases, and intradetrusor Onabotulinumtoxin-A (BTX-A) injection over the past 6 months. Patients with prior slings (midurethral or pubovaginal), bladder/bladder neck suspension operations, or previous bladder reconstruction procedures such as augmentation cystoplasty will also be excluded. Patients with active UTI can be treated and subsequently screened for the trial. Patients with history of intradetrusor BTX-A could be screened 6 months post-injection. For non-urologic conditions, botulinum toxin is permitted. Patients taking bladder medications (anticholinergics, beta-agonists, or alpha-blockers) at study entry will continue to take them throughout the study, and those not taking them are to remain off of them throughout the study. Table 1 detailed the inclusion and exclusion criteria.

**Table 1:** Inclusion and exclusion criteria

Inclusion	Exclusion
• Adult female (≥ 18 years old) • Clinically stable MS (EDSS ≤ 6.5) • Bladder symptoms ≥ 3 months • VD diagnosis, defined as o %PVR/BC > 40%, OR o Liverpool Nomogram percentile < 10%, OR o Perform self-catheterization	• Male • Severe debilitating MS • History of recent seizure (< 1 year) • Pregnant or planning to become pregnant • Contraindication to MRI • History of augmentation cystoplasty • Recent BTX-A injection (< 6 months) • Other neurological disorders beside MS

### Power analysis

Although fMRI studies suggest that approximately 12–20 subjects are required to achieve 80% power at the single voxel level for typical activations, while accounting for intra- and inter-subject variability [21, 22], most published fMRI studies specifically evaluating bladder function have included 8–12 subjects. For this pilot study, we propose an enrollment goal of 10 subjects to ensure a well-powered study and account for exclusion due to movement artifact. This sample size will be used to calculate the effect size for our feasibility outcomes analysis.

### Planned interventions

**Fig. 1:**
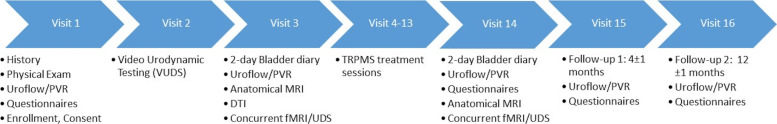
Schematic representation of clinic visits where data will be collected and patients will receive treatment

Subjects will undergo 16 visits in total throughout the entire duration of the study. Details of each clinic visit are specified in Fig. 1.

#### Baseline evaluations (visit 1 and 2)

During screening visit, our research coordinator will explain the study in details to subjects, and answer any questions they may have related to the study. Subjects will be given sufficient time to privately review the study material and Informed Consent Form before consenting to participate.

At screening (visit 1), each subject will provide a detailed history and undergo a complete physical examination. Each subject will have the following assessments: History and Physical Exam, Expanded Disability Status Scale (EDSS), American Urological Association Symptom Score (AUASS), Neurogenic Bladder Symptom Score (NBSS), Urogenital Distress Inventory (UDI-6), Incontinence Impact Questionnaire (IIQ-7), Hospital Anxiety and Depression Scale (HADS), Hamilton Depression Rating Scale (HAM-D), Hamilton Anxiety Rating Scale (HAM-A), and MRI Safety Screening Questionnaire. Two-day bladder diary, non-instrumented uroflow, and post-void residual (PVR) volume will be measured and collected. A urine sample will be obtained for urinalysis and pregnancy (if applicable). After subjects are screened and consented (visit 1), a clinical video UDS (VUDS) will be performed (visit 2). If subjects had a VUDS report within 1 year prior to the study, this VUDS will be accepted for the study and subjects will not undergo an additional VUDS during visit 2. Subjects have the option to combine visit 1 (screening) and visit 2 (VUDS).

#### Baseline scan: concurrent fMRI/UDS examination (visit 3)

Prior to the scan, subjects will receive a treatment cap-fitting session where their head measurement will be recorded to identify the 10–20 EEG position marking [23]. Five fiduciary pills will be placed at the F3, F4, C3, C4, and Cz locations to aid the ROI identification. The study procedure will be explained to the subjects, and they will be asked to completely empty their bladder before entering the MRI scanner. A 7-Tesla Siemens MAGNETOM Terra MRI scanner with 32 channel single transmit head coil will be used (3T Siemens MAGENTOM Vida MRI scanner will be used in subjects with contraindications for 7T scanner).

**Fig. 2:**
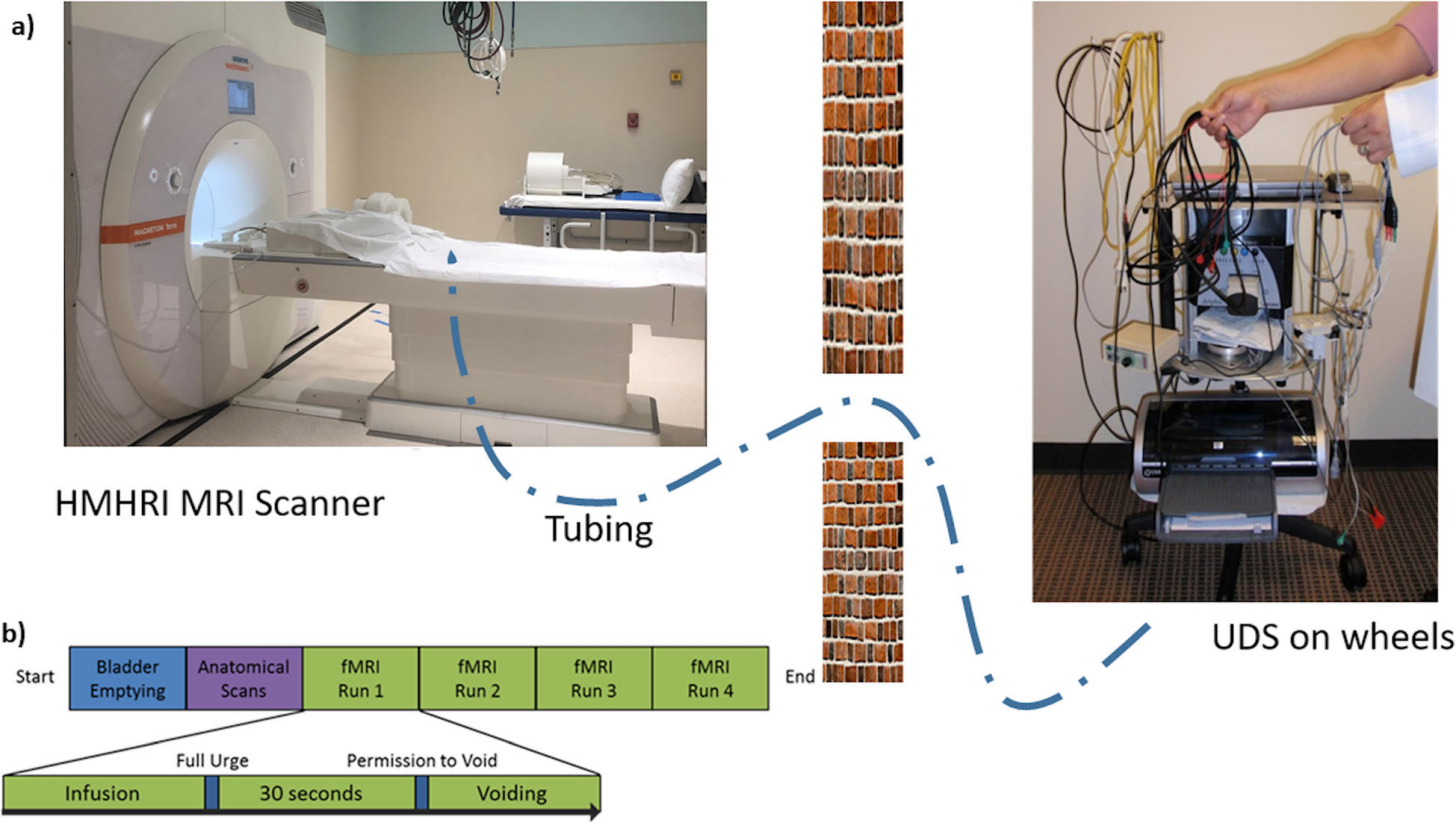
**a** Concurrent fMRI/UDS setup where MRI-compatible bladder and rectal catheters are passed through a small opening in the wall between the control and scanner room. **b** Concurrent fMRI/UDS testing protocol

When in the scanner, double lumen 7Fr MRI-compatible UDS bladder and rectal catheters will be placed in the subjects and the tubing will be extended out to the control room to record abdominal, vesical, and detrusor pressures via a UDS machine (Fig. 2a). Subjects will be instructed to use a button placed in their right hand to indicate the time of full urgency (“strong desire to void”), when they start voiding, and when they finish voiding. UDS machine will record when subjects start voiding and monitor the entire bladder cycle.

Three dimensional structural images will be obtained from a T1-weighted MPRAGE sequence; (sagittal direction, 0.7 mm-isotropic resolution). Diffusion Tensor Imaging (DTI) images will be acquired (64 directions, five B0 images) on the scanner. Functional images will be collected afterwards using simultaneous UDS analysis (axial echo-planar, TR = 2500 ms, 1.4 mm slice thickness, 1.4 mm in-plane resolution).

During the concurrent fMRI/UDS, the bladder will be gradually filled with room-temperature sterile saline at 50 mL/min until subjects signaled a “strong desire to void.” Subjects will then be instructed to hold for 30 s, after which they will be given permission to void. UDS will be performed at the same time to monitor the entire filling and voiding cycles. After voiding or an attempt to void was completed, bladder will be drained to ensure each cycle started with an empty bladder, and the cycle was repeated three to four times depending on subjects’ tolerance (Fig. 2b). Care will be taken not to exceed 45 min for the total duration of the fMRI examination.

#### Intervention: transcranial rotating permanent magnet stimulator

**Fig. 3:**
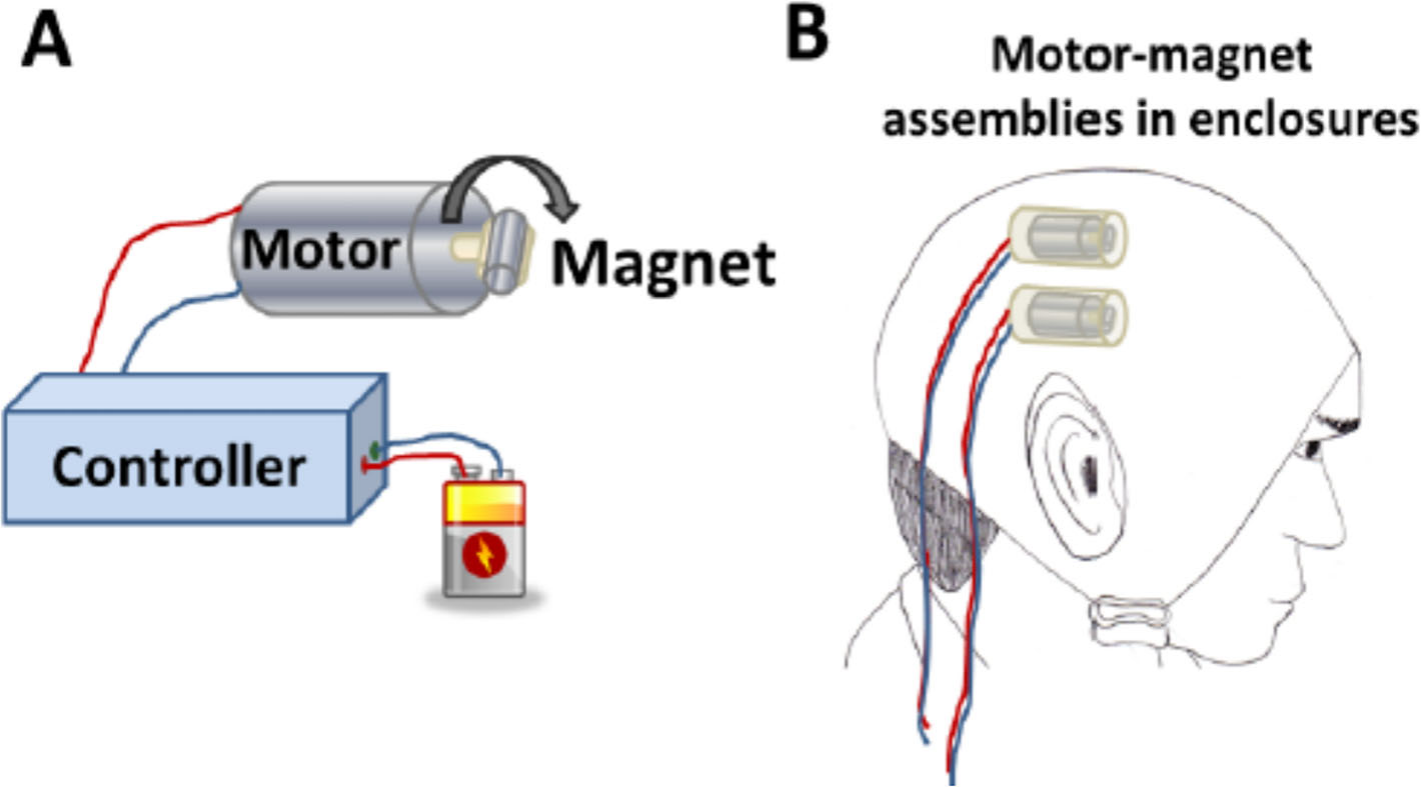
TRPMS cap with microstimulators placed on sites corresponding to left primary motor, right lateral premotor, and right supplementary motor cortex

The TRPMS device used in this study consists of axially magnetized cylindrical magnets attached to, and rapidly rotated by, battery-operated DC motors (Fig. 3). The magnet-motor assemblies called microstimulators generate an oscillating magnetic field whose effective strength for neuronal stimulation can penetrate a depth of ~ 2 cm corresponding to the depth of the cerebral cortex from the surface of the scalp [24]. The TRPMS microstimulators generate specially devised repeated oscillating magnetic field patterns that induce electric currents in the cerebral cortex by electromagnetic induction. These currents depolarize the cell membrane of cortical neurons, and either excite or inhibit them, depending on the stimulus parameters, such as pulse duration, inter-pulse interval, and total duration of stimulation [7].

Multiple TRPMS microstimulators can be used simultaneously to produce patterned multifocal stimulation [24]. TRPMS can therefore selectively induce, modulate, or suppress neuronal activity, and also modulate strengths of functional connections between two or more cerebral cortical areas when they are stimulated simultaneously, depending on the strength, frequency, and pattern of stimulation [7].

Up to 6 microstimulators can be attached at desired locations to a Neoprene cap worn on the head to stimulate desired cortical locations. The neoprene cap prevents the microstimulator(s) from coming in direct contact with the scalp and is individualized for each patient based on their brain anatomy. The microstimulators are connected by a cable to, and activated by, a stimulator console consisting of an electronic circuit and a microprocessor. A prescribed program uploaded in this portable stimulator console will deliver the desired magnetic stimulation to the brain. The stimulator is turned on and off by a manual switch.

TRPMS is determined a non-significant risk device by the Food and Drug Administration (FDA) (Fig. 3).

#### TRPMS treatment setup

Based on data found in the literature, we selected specific cortical brain regions that are known to be involved during the micturition cycle, and refined the list to come up with the following template (Table 2) for regions to modulate (stimulate/inhibit) in this pilot clinical trial.

**Table 2:** Cortical regions to modulate and their corresponding tasks

Cortical regions	Task	Stimulated/inhibited?
Right inferior frontal gyrus (IFG)	Voiding initiation	Stimulated (40 min)
Left dorsolateral prefrontal cortex (dlPFC)	Depression mitigation	Stimulated (40 min)
Bilateral supplementary motor area (SMA)	Pelvic floor contraction	Inhibited (10 min)
Right middle frontal gyrus (MFG)	Pelvic floor contraction	Inhibited (10 min)
Right dorsolateral prefrontal cortex (dlPFC)	Anxiety	Inhibited (10 min)

These regions will be identified for each patient based on their anatomy and activation, derived from patients’ anatomical and functional MRI scans, respectively. The Montreal Neurological Institute (MNI) coordinates of these regions based on their activation/deactivation will be recorded, from which their corresponding 10–20 EEG positions will be obtained. The target sites of modulation on the neoprene treatment cap and subsequently the microstimulators’ locations will then be marked on the cap using the identified 10–20 EEG positions derived from patients’ head measurement, and five fiduciary vitamin E capsules attached to the cap in relation to the patients’ brain anatomy and activation on their MRI brain scan. The microstimulators will be securely attached at target sites onto the treatment cap that the patient will wear during each treatment session. The treatment, therefore, will be individualized for each patient and consistent throughout the entire treatment course.

Each microstimulator can be independently programmed to deliver stimulation to either excite or inhibit neurons at targeted cortical sites. The portable stimulator console will be connected to the microstimulators and a rechargeable battery powering it. In the current treatment for this study, inhibitory stimulation will be achieved by using 100 ms pulse train duration (TD) repeated at a train interval (TI) of 5 s for 10 min [25]. Excitatory stimulation will be produced with the same TD and TI, but with total duration of stimulation lasting 40 min. The total duration of one treatment session, therefore, will be 40 min [25].

#### TRPMS treatment (visit 4–13)

Patients will receive 10 TRPMS treatment sessions in the clinic for 2 weeks (visit 4–13). Each treatment session will last for a total of 40 min, with continuous supervision by a study team member for the entire duration of each session. Patients will fill out a set of questionnaires before and after each treatment session to identify any potential adverse event related to TRPMS.

#### Post-treatment (visit 14) and follow-up assessments (visit 15 and 16)

Within 2 weeks after the completion of the TRPMS treatment, patients will return to the clinic for a post-treatment visit (visit 14), where non-instrumented uroflow and PVR of patients will be measured, and validated questionnaires and 2-day bladder diary will be collected. At this visit, patients will also repeat the fMRI/UDS examination that they received at baseline scan visit (visit 3).

Patients will return for two follow-ups at 4 ± 1 months (visit 16) and 12 ± 1 months (visit 17) following treatment where non-instrumented uroflow, PVR, and questionnaires will be measured and collected.

### Feasibility outcome measures

TRPMS trials will be determined feasible if ≥ 20% recruitment rate and ≥ 80% retention rate are achieved within the proposed study timeline (3 years), and an effect size of approximately 0.8 is confirmed with the proposed enrollment goal (10 patients) for this pilot study.

### Study outcome measures

The primary outcomes are BOLD activation and FC patterns of patients at baseline and following treatment. Increased in the BOLD signal activation of a brain region indicates more blood flow to that region, suggesting more neural activity during a specific task, such as tasks during the bladder cycle. Functional connectivity (FC) is the connectivity between brain regions that share functional properties. Task-based FC (such as FC during voiding initiation) is suggested to be an expression of the network behavior underlying brain function. Since MS lesion locations may affect not only the regions of interest but they may also interfere with communication between these regions, investigating the FC pattern could yield meaningful implications in evaluating VD in MS.

The secondary outcomes include both objective clinical variables (2-day bladder diary, non-instrumented uroflow variables, and PVR obtained using bladder scanner) and subjective clinical variables (validated questionnaires). These will be used to assess any clinical improvement of patients’ VD symptoms.

The tertiary outcomes are the baseline factors that could predict response to treatment. Responders to treatment will be identified as those with at least one of the following criteria:

- %PVR/BC ≤ 20% post-treatment and ≥ 40% at baseline [26].
- %PVR/BC decreased by at least 50% of baseline %PVR/BC value [26].
- Liverpool nomogram percentile ≥ 25% post-treatment and < 10% at baseline [27].

Since either %PVR/BC or Liverpool nomogram percentile is used as an inclusion criteria, they will be used to define responders to treatment. Because the study population is MS patients with NLUTD, response to treatment is not defined as the same values observed in healthy individuals with normal voiding, but rather values that have been observed in patients with neurogenic bladder without VD diagnosis [26, 27].

### Statistical analysis

#### Neuroimaging data analysis

AFNI software will be used for analysis. Structural and functional images will be registered and motion-corrected. Patients with rapid motion will be excluded. Voxel activation will be identified at the time of “voiding initiation.” Significant activated voxels will be identified at “voiding initiation” under generalized linear model (GLM). Group level analysis will be performed by transforming data into MNI space, and significantly activated voxels will be identified using a Student’s *t* test. Comparisons will be drawn between baseline and post-TRPMS scans of patients.

Functional connectivity (FC) analysis will be performed using AFNI. fMRI images acquired during the voiding task together with their corresponding anatomical datasets will be processed using the default-mni preprocessing option to enable group analysis in MNI space. A region-based connectivity analysis will be performed for patient group at baseline and post-treatment based on regions of interest (ROI, *n* = 4) defined in a brain atlas available from the toolbox. FC is the connectivity between brain regions that share functional properties. It can be defined as the temporal correlation between spatially remote neurophysiological events assessed by their respective BOLD signal time courses. FC will be quantified by *T* values for a *p* value < 0.05 (two-sided, FDR-corrected).

The baseline DTI images will be transferred to on offline workstation for further processing. The software packages TrackVis (version 0.6.0.1) and the Diffusion Toolkit (version 0.6.3, trackvis.org) will be used to extract selected white matter tracts (WMT) of interest and their fractional anisotropy (FA) and mean diffusivity (MD) values. These WMTs and values will be used to assess whether the integrity of any WMT is found to have contributions to identifying level of responses to TRPMS treatment.

#### Clinical data analysis

Clinical subjective variables (validated questionnaire answers) and objective variables (non-instrumented uroflow data) will be compared between baseline and post-treatment using paired Student’s *t* test (or Wilcoxon test if sample is determined to be non-parametric). Baseline values will also be compared with these clinical data collected at 4-month and 12-month follow-ups. RStudio (Version 1.2) will be used to perform statistical analysis.

#### Sub-analysis for responders to treatment

Analysis of covariance (ANCOVA) will be used to assess the difference in clinical/UDS data between responders and non-responders group, with baseline neuroimaging and UDS values as covariates, and CIC-dependence, additional presence of storage phase symptoms, detrusor sphincter dyssynergia, and occurrence of detrusor overactivity during concurrent fMRI/UDS as factors. Additional post hoc analyses of adverse events will also be performed.

#### Feasibility outcomes analysis

The feasibility outcome measures will be calculated and analyzed as follows:

- Recruitment rate: calculated by dividing the number of enrolled participants by number of patients screened for eligibility from our clinic and advertisement resources.
- Retention rate: measured as percentage of participants completing the study through the entire 16 visits.
- Effect size: confirmed post hoc using Cohen’s *d* value, calculated using patients’ %PVR/BC at baseline and post-treatment.

### Safety assessment and adverse event management

Every subject will be screened with a urine pregnancy test at enrollment. Subjects with a positive pregnancy test will be excluded from the study. All subjects undergoing UDS will be screened for UTI prior to testing. Subjects will be given information regarding the symptoms and signs of a UTI and asked to call the urology investigator’s office if they have any of the symptoms after testing. If a UTI is present, it will be treated. Subjects will be screened for MRI safety using TMH standard screening forms. Before proceeding with fMRI testing, subjects will be asked to remove all clothing and items containing metal. All materials used during the fMRI/UDS scans are MRI compatible.

Before the start of each treatment session, subjects will fill out a safety questionnaire regarding any abnormal symptoms regarding their feelings, mood, pain, and sensation that they might experience after the completion of the previous treatment session. After the treatment session is completed, they will fill out the same questionnaire to confirm if they experience any abnormal symptoms during the treatment session. This will be used to assess any treatment-related adverse effects to participants. Each treatment session will also be closely monitored by a study team member at all time during the course of the treatment.

During the course of the study, subjects are responsible to notify the study team of any adverse events (AEs) or side effects that they experience. If there is a serious AE during the treatment course, such as seizure, the subjects will be removed immediately from the treatment, and Houston Methodist Rapid Response Team will be contacted immediately to resolve any life-threatening situation. If there is any clinically significant AE evaluated by the PI, or any medical condition or situation occurs such that continued participation in the study would not be in the best interest of the subjects, they will be withdrawn from the study. Subjects who withdraw as a consequence of AEs will be followed up by the study for 3 months, and effort will be made to undertake safety follow-up procedures under medical supervision until the symptoms of any AE resolve or the subject’s condition becomes stable.

### Lost to follow-up

Subjects who are enrolled in the study will be kept in close communication by our study coordinator to ensure strict adherence to the study schedule. During the study, subjects who are lost to follow-up will be contacted by phone and subsequently through their general practitioner to obtain reasons for their deviation. Records relating to all patients lost to follow-up will be retained, and analyses will proceed on an intent-to-treat basis.

### Data and safety monitoring

Appropriate measures will be taken by the study team to ensure confidentiality of participants’ identity and private health information. Study information for each patient will be recorded and stored in a locked cabinet and a Houston Methodist-registered laptop dedicated for research, both are stored securely within our facility. Only our research team will have access to this information.

All members of the study team will stay current with training in the protection of human research participants and HIPAA. Institutional Research Board (IRB) approval has been obtained for this study by Houston Methodist Research Institute (HMRI). The principal investigator (PI) will regularly monitor the data in collaboration with the research team, with periodic review by HMRI IRB. Further, we will minimize risks by using rigorous data security protocols to protect subject confidentiality and comply with all federal and HIPAA requirements. The study team will identify protocol deviations (e.g., violation of human subject confidentiality or comprised data integrity) and will ensure expedient reporting to the IRB.

This study will utilize the HMRI Data Review Committee (DRC) as a resource for data and safety monitoring. The HMRI-DRC will perform data monitoring on a regular basis, at least once a year. Clinical safety data including the following will be evaluated:

- Overall protocol accrual and expected number of patients to be treated.
- Patient registrations with regard to eligibility.
- All adverse events and their relationship to the protocol therapy.
- Response evaluations.
- Whether protocol-specific rules are being followed.
- Study amendments/modifications that may have occurred since the last review.

### Ethics and dissemination

All subjects will be properly counseled and consented before enrolling in this study. No minor or vulnerable individuals will be recruited for the study. Our informed consent states that participation is completely voluntary, and subjects can withdraw at any point and this will not affect their relationship with the physician and their treatment course. All staff members involved in the collection of data and handling of patients will have proper privileges and training by our Research Institute and Translational Imaging Center.

All data and resources developed within the scope of this proposal will be made available to the scientific community. This includes publications (through PubMed Central and/or publication in open-access journals) and raw data if applicable.

## Discussion

With the evolution of neuroimaging tools and resources, such as fMRI, we have begun to gain additional insights into brain control over bladder function in both healthy and patients with neurogenic bladder dysfunction. Despite the central role of the central nervous system in urinary control and its disruption secondary to MS, there has been no treatment for neurogenic bladder dysfunction by targeting the brain centers that are involved in normal bladder function. Cortical modulation through the use of TRPMS is a valuable alternative to address these symptoms and improve the quality of life for MS patients. By directly targeting the central nervous system (the source cause of symptoms) using a non-invasive and individualized procedure (based on each patients’ brain) to facilitate anatomical cortical differences, TRPMS may provide a new therapeutic option for symptomatic patients.

Some potential limitations of this study include the supine position during the fMRI/UDS examination, which could pose difficulty for subjects to void in the scanner. MS is a heterogeneous disease, and the observed changes in outcomes could be a result of historical changes unrelated to treatment, or depending on baseline factors such as location of lesion burdens or duration of MS. However, we plan to perform sub-analyses to determine which of these factors could affect the study results or become independent predictors for treatment outcomes.

This study, to our knowledge, will be the first of its kind in humans to consider non-invasive and individualized cortical modulation for treating VD in MS patients. The data and results obtained from this study will broaden our current knowledge of brain control of the bladder and the brain-level indicators for progression of VD in MS patients to give us a much better understanding of the uro-neurological manifestations of this complex disorder. This new understanding of the superficial regions of the brain related to voiding and response to therapy might shift clinical attention from the end organ to more cerebral control, possibly creating further avenues for intervention that could transform management of NLUTD in MS. Lastly, this study will encourage and provide the foundation for similar research in other neurogenic bladder pathologies, such as spinal cord injury, stroke, and Parkinson’s disease.

## Trial status

Trial registration: ClinicalTrials.Gov, NCT03574610. Registered 2 July 2018, https://clinicaltrials.gov/ct2/show/NCT03574610. Full approval has been obtained from the Institutional board review (IRB) at Houston Methodist Research Institute (PRO00019329, Version 1, approved on 15 May 2018). Patient recruitment started in July 2019, and is expected to be completed by July 2021. The trial is currently ongoing. The protocol reported in this manuscript is based on PRO00019329, Version 5.0, approved on 23 April 2020.

## Data Availability

On request and based on approval from relevant authorities, any data required to support the protocol can be provided. Dr. Rose Khavari will have access to the final trial dataset.

## Abbreviations

AE: Adverse event
BC: Bladder capacity
BOLD: Blood-oxygen-level-dependent
BTX-A: Onabotulinumtoxin-A
CIC: Clean intermittent catheterization
EDSS: Expanded disability status score
EEG: Electroencephalography
FA: Fractional anisotropy
FC: Functional connectivity
fMRI: Functional magnetic resonance imaging
HMRI: Houston Methodist Research Institute
IRB: Institutional Review Board
MD: Mean diffusivity
MNI: Montreal Neurological Institute
MRI: Magnetic resonance imaging
MS: Multiple sclerosis
NLUTD: Neurogenic lower urinary tract dysfunction
PAG: Periaqueductal grey
PMC: Pontine Micturition Center
PVR: Post-void residual
ROI: Region of interest
TD: Train duration
TI: Train interval
TR: Repetition time
TMS: Transcranial magnetic stimulation
TRPMS: Transcranial rotating permanent magnet stimulator
UDS: Urodynamic study
UTI: Urinary tract infection
VD: Voiding dysfunction
WMT: White matter tract
